# Bioaccumulation of Microplastics in Decedent Human Brains Assessed by Pyrolysis Gas Chromatography-Mass Spectrometry

**DOI:** 10.21203/rs.3.rs-4345687/v1

**Published:** 2024-05-06

**Authors:** Matthew Campen, Alexander Nihart, Marcus Garcia, Rui Liu, Marian Olewine, Eliseo Castillo, Barry Bleske, Justin Scott, Tamara Howard, Jorge Gonzalez-Estrella, Natalie Adolphi, Daniel Gallego, Eliane El Hayek

**Affiliations:** University of New Mexico; University of New Mexico; University of New Mexico; University of New Mexico; University of New Mexico; University of New Mexico; University of New Mexico; Oklahoma State University; University of New Mexico Health Sciences Center; Oklahoma State University; New Mexico Office of the Medical Investigator; New Mexico Office of the Medical Investigator; University of New Mexico

**Keywords:** Polymer, neuronal, autopsy, liver, kidney, nanoplastics

## Abstract

Rising global concentrations of environmental micro- and nanoplastics (MNPs) drive concerns for human exposure and health outcomes. Applying pyrolysis gas chromatography-mass spectrometry (Py-GC/MS) methods to isolate and quantify MNPs from human samples, we compared MNP accumulation in kidneys, livers, and brains. Autopsy samples from the Office of the Medical Investigator in Albuquerque, NM, collected in 2016 and in 2024, were digested for Py-GC/MS analysis of 12 polymers. Brains exhibited higher concentrations of MNPs than liver or kidney samples. All organs exhibited significant increases from 2016 to 2024. Polyethylene was the predominant polymer; the relative proportion of polyethylene MNPs was greater in brain samples than in liver or kidney. Transmission electron microscopy verified the nanoscale nature of isolated particles, which largely appeared to be aged, shard-like plastics remnants across a wide range of sizes. Results demonstrate that MNPs are selectively accumulated into the human brain and concentrations are rising over time.

The ubiquitous presence of plastics, especially polymer-derived particulates ranging from 500 micrometers in diameter down to 1 nanometer, defined as micro- and nanoplastics (MNP), is a defining feature of the Anthropocene epoch^1^. The extent to which microplastics cause harm or toxicity is unclear, although recent studies associated MNP presence in carotid atheromas with increased inflammation and risk of future adverse cardiovascular events^2,3^. In controlled exposure studies, MNPs clearly enhance or drive toxic outcomes^4–6^. The mantra of the field of toxicology – “dose makes the poison” (Paracelsus) – renders such discoveries as easily anticipated; what is not clearly understood is the internal dose in humans.

To date, several studies have utilized visualization and spectroscopic methods to identify and count particulates in organs such as the lungs, intestine^7^, and placenta^8^. These methods are often limited to larger (>1–5μm) particulates, thus nanoplastics are excluded from the quantitation. As a novel approach, pyrolysis gas chromatography-mass spectrometry (Py-GC/MS) has been applied to blood^9^, placentas^10^ and recently major blood vessels^2,3^ in a manner that appears more cumulative and quantitative, and less biased than visual identification methods. Py-GC/MS data between labs has been comparable, providing confidence in this method for human tissue analysis^2,9,10^. We applied Py-GC/MS to assess the relative distribution of MNPs in major organ systems from human decedent livers, kidneys, and brains.

## METHODS

### Human Tissue Samples:

We obtained de-identified, post-mortem human liver, kidney, and brain (frontal cortex) samples, retrospectively, in cooperation with and approval from the University of New Mexico Office of the Medical Investigator (OMI) in Albuquerque, New Mexico, under the guidance of a trained forensic pathologist (DFG) who selected consistent regions from all organs. Samples were available from 2016 and 2024; the same collection protocol was used for 2016 and 2024. Small pieces of representative organs (3 to 5 cm^2^) are routinely collected at autopsy and placed in a small container with 10% formalin. Limited demographic data was available due to the conditions of specimen approval. In the 2016 samples, 17 samples were from males and 10 were from females. In 2024, 13 samples were from males and 11 were from females. The mean (and standard deviation) age of 2016 decedents was 50.0 (±11.4) years and 52.3 (±16.8) years for the 2024 decedents.

### Py-GC/MS Detection of Polymer Solids:

Formalin-fixed tissue samples (approximately 500mg) were digested with 10% potassium hydroxide for 3d at 40°C with intermittent manual mixing to ensure even and thorough digestion. Fully digested samples were then ultracentrifuged at 100,000g × 4h to generate a pellet enriched in solid materials resistant to such digestion, principally polymer-based solids^10^. A 1–2 mg portion of the resulting pellet was then analyzed by single-shot Py-GC/MS and compared to a microplastics-CaCO_3_ standard containing 12 specific polymers: Polyethylene (PE), Polyvinyl chloride (PVC), Nylon 66 (N66), Styrene-butadiene (SBR), Acrylonitrile Butadiene Styrene (ABS), Polyethylene terephthalate (PET), Nylon 6 (N6), Poly(methyl methacrylate) (PMMA), Polyurethane (PU), Polycarbonate (PC), Polypropylene (PP), Polystyrene (PS). Polymer spectra were identified via the F-Search MPs v2.1 software (Frontier Labs). Resulting data were normalized to original sample weight to render a mass concentration (μg/g).

### Data Analysis:

Statistical analysis was performed using GraphPad Prism v10.0.03. Details of statistical analysis are provided in the data supplement.

## RESULTS and DISCUSSION

**Figure 1:**
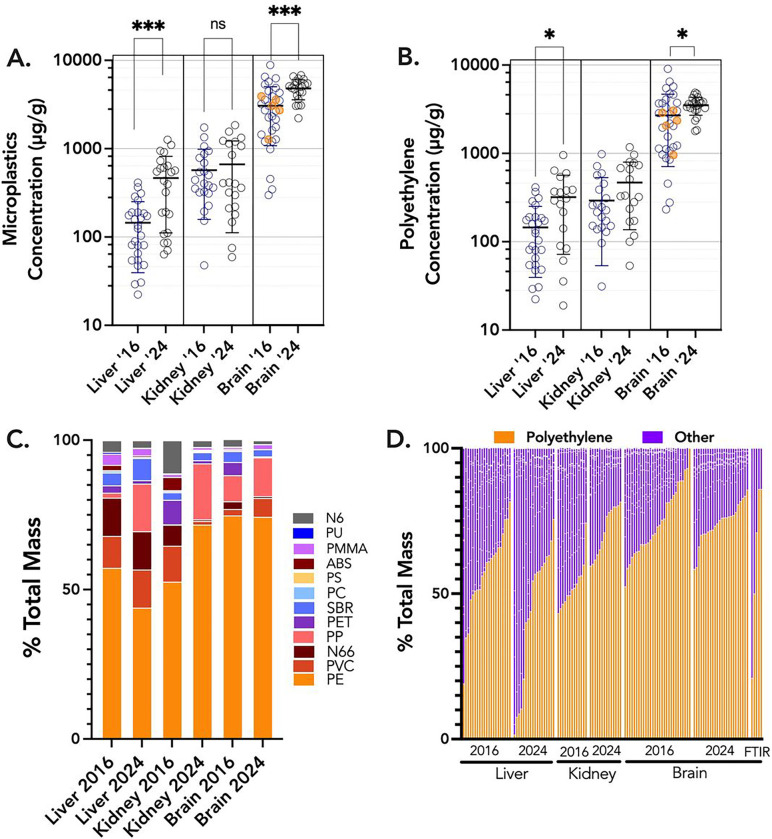
Overview of total MNP concentrations from all decedent samples from liver, kidney, and brain. **A.** All data shown, with the bar representing arithmetic mean value and the standard deviation. Orange colored symbols in the 2016 brain samples were analyzed independently at Oklahoma State University. Asterisks indicate significant differences temporal changes (from 2016 to 2024) using a nonparametric t-test (Mann Whitney). Brain concentrations were also significantly higher than liver and kidney, by ANOVA. **B.** Using only polyethylene data, similar trends were noted, although the kidney concentrations did not increase in the 2024 samples. **C.** Overall distribution of 12 different polymers suggests a greater accumulation of polyethylene in the brain relative to liver or kidney. Polyethylene (PE), Polyvinyl chloride (PVC), Nylon 66 (N66), Styrene-butadiene (SBR), Acrylonitrile Butadiene Styrene (ABS), Polyethylene terephthalate (PET), Nylon 6 (N6), Poly(methyl methacrylate) (PMMA), Polyurethane (PU), Polycarbonate (PC), Polypropylene (PP), Polystyrene (PS). **D.** Distribution trends for PE across each organ and collection date, including 5 additional samples (on the right) from the 2016 brain collections that were analysed by Attenuated Total Reflectance-Fourier-transform infrared spectroscopy (FTIR).

Py-GC/MS has proven to be an informative and reliable method to determine plastics concentrations in liquid and solid tissue samples, with ample assurance of accuracy, quality, and rigor^2,3,9,10^. Decedent liver and kidney MNP concentrations were similar, with means of 465 and 666 μg/g, respectively, from 2024 samples (Figure 1A). These were higher than previously published data for human placentas (126 μg/g)^10^, but comparable to testes (329 μg/g)^11^. Liver samples had significantly higher concentrations in 2024 than in 2016 samples (145 μg/g; p<0.001). The brain samples, all derived from the frontal cortex, revealed substantially higher concentrations than liver or kidney, at 3,057 μg/g in 2016 samples and 4,806 μg/g (0.48%, by weight) in 2024 samples, ranging as high as 8,861 μg/g. Five brain samples from 2016 (highlighted in orange, Figure 1A,B) were analyzed independently by colleagues at Oklahoma State University, and those values were consistent with our findings.

A non-parametric analysis of variance (Kruskal-Wallis) confirmed that MNP concentrations in brains were significantly greater than all other tissues (P<0.0001). Furthermore, from 2016 to 2024, there was a significant increase in MNP concentrations in both livers and brains. The predominant polymer found in all tissues was polyethylene, which independently displayed similarly increasing trends from 2016 to 2024 in the liver and brain (Figure 1B). The proportion of polyethylene in the brain (74%) appeared significantly greater relative to other polymers in comparison to the liver and kidney (44–57%), although kidney samples from 2024 also had increased relative PE (71%; Figure 1C,D). This was also confirmed with ATR-FTIR spectroscopic analysis from 5 brain samples (Figure 1D).

**Figure 2:**
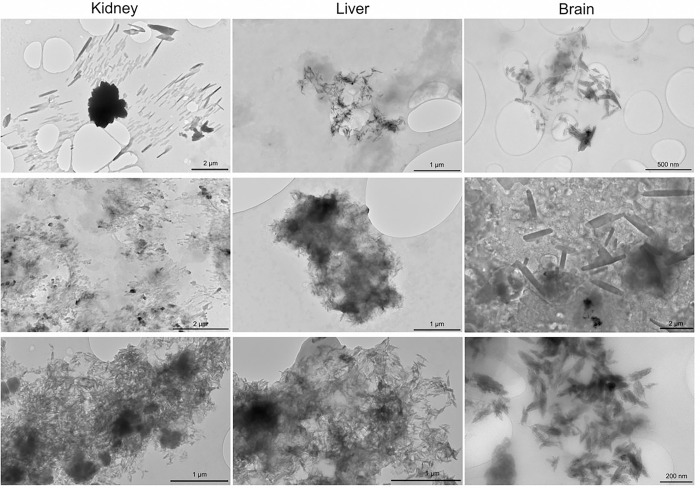
Example TEM images of solid nanoparticulates derived from kidney (left), liver (center), and brain (right) samples. While TEM does not permit spectroscopic identification of particulate molecular composition, the bulk of particulates that were predominantly polymer as assessed by ATR-FTIR appear to be of these sizes and shapes. Shard-like appearances, with dimensions ranging from micrometer to nanometer sizes, suggest an aged, friable polymer composition.

Because we suspected that much of the MNPs measured were actually in the nanoscale range, transmission electron microscopy (TEM) was conducted on the dispersed pellets obtained from kidney, liver, and brain (Figure 2; see methods supplement). While TEM does not provide spectroscopic identification to confirm particulate composition, we observed common shapes and sizes among the numerous samples and tissue types. Notably, there were innumerable particulates with shard-like appearance, often less than 200 nm in length. Currently, MNP uptake and distribution pathways are incompletely understood; this new appreciation of the size and shape aids in our appreciation of potential mechanisms. Importantly, these observations bring into question the relevance of the many recent studies utilizing polystyrene microspheres^4,12^, as polystyrene was infrequently detected in human tissues and MNPs were rarely spherical.

The concentrations in liver and kidney were not as high (relative to brains) as we would have suspected, as these are “front line” organs for xenobiotic uptake and clearance. That said, the lipophilic nature of plastics may make them easily handled by the liver, which has a major role in uptake and repackaging of dietary triglycerides and cholesterol. A recent study found higher MNP numbers in the cirrhotic liver compared to the healthy liver; whether the microplastics promote disease or are simply accumulating along with intracellular fats has not been elucidated^13^.

Following this logic, the human brain has the second highest lipid content in the body, with only adipose tissue being higher; brain MNP concentrations are comparable to recently published Py-GC/MS data from carotid plaques, which are also a lipid depot^3^. Furthermore, the brain receives a high blood flow, approximately 25–30% of the cardiac output, and has a tremendous metabolism. The blood-brain barrier poses a notorious challenge. However, modeling of transfer across cellular membranes suggests the uptake is dependent on the association of particulates with cholesterol and, furthermore, that particles <1μm rapidly traversed the blood-brain barrier within 2h of ingestion in mice^14^. Longer-term gavage studies similarly found that larger (5 μm) polystyrene microspheres could access the brain and promote metabolomic alterations^15^. Lastly, clearance rates from the brain are unknown for polymer particulates. The lack of correlation with the decedent age suggests that an equilibrium occurs and may depend on genetic, dietary, and lifestyle factors that ultimately contribute to the wide between-subject variability in MNP concentrations. In zebrafish exposed to constant concentrations, nanoplastics uptake increased to a stable plateau and cleared after exposure^16^; however, the maximal concentrations were increased proportionately with higher exposure concentrations. While the time course for kinetics is assuredly longer in humans, we postulate that the exponentially increasing environmental concentrations of MNPs^1,17^ will analogously increase internal maximal concentrations, which is corroborated by our finding that total plastics mass concentration in brains increased over 50% in the past 8 years.

## LIMITATIONS

The present data are derived from novel analytical chemistry methods that have yet to be widely adopted and refined. Several quality control steps ensure that external contaminants are not incorporated into the sample calculations, including KOH blank samples and measurement of the polymer composition of all plastic tubes and pipette tips that are essential in the digestion and measurement process. Notably, given the consistent nature of handling and processing across varying organ samples (*i.e*., brain, liver, kidney), the dramatic, selective accumulation of MNPs in the brain cannot be dismissed as an artefact of contamination. Furthermore, the far longer duration of samples in plastic stock jars from 2016 (84–96 months) compared to those samples from 2024 (1–3 months) and the significantly lower plastics content in 2016 samples suggests that contamination from fresh plastics is not a concern to the conclusions from these data.

Both laboratories (UNM and OSU) observed a within-sample coefficient of variation of approximately 25%. This does not alter the conclusions regarding the temporal trends of selective accumulation in brains, given the magnitude of those effects. However, we believe several steps may be valuable to improve the precision of Py-GC/MS output, which in turn should improve assessment of health outcomes for future studies. There may be value in limiting assessments to the nanoscale range, which could incorporate longer ultracentrifugation times as well as a filtration of >1 μm particulates. Ambient air particulate matter research provides some justification that “smaller is worse”, which led to the transition from air quality standards based on particles <10 μm in diameter to those <2.5 μm, which aligned more closely with health outcomes^18^. Additionally, the Py-GC/MS method is limited to small sample weights (~1–2mg), which presents challenges for sampling and weighing accuracy when even small portions of tissue (~500 mg) generate large polymer-containing pellets; however, larger sample sizes may not be feasible due to the rapid combustion required for this approach. Lastly, by obtaining only a single sample from each organ for each subject, distribution heterogeneity within tissues remains uncharacterized.

## CONCLUSIONS

MNP concentrations in decedent brain samples ranged from 7-to-30 times the concentrations seen in livers or kidneys. With independent confirmation from another laboratory and visual evidence from FTIR and TEM approaches, we have high confidence that MNPs selectively accumulate in the brain, with the majority being nanometer-scale, shard-like particulates. However, linking MNP concentration data to health outcomes in larger cohorts will require refinements to the technique, more complex study designs, and larger cohorts. The parallels between the present data showing an increasing trend in MNP concentrations in the brain with exponentially rising environmental presence of microplastics^19–21^ and increasing global rates of age-corrected Alzheimer’s disease and related dementia^22–25^, given the potential role of anionic nanoplastics in protein aggregation^26^, add urgency to understanding the impacts of MNP on human health.
